# Highly Active Antiretroviral Therapy (HAART)-Related Hypertriglyceridemia Is Associated With Failure of Recovery of CD14^low^CD16^+^ Monocyte Subsets in AIDS Patients

**DOI:** 10.1097/MD.0000000000001115

**Published:** 2015-07-13

**Authors:** Junyan Han, Hongxin Zhao, Yaluan Ma, Haiwei Zhou, Yu Hao, Yanmei Li, Chuan Song, Ning Han, Xiangyi Liu, Hui Zeng, Mingzhao Qin

**Affiliations:** From the Institute of Infectious Diseases, Beijing Ditan Hospital, Capital Medical University (JH, HZ, YH, YL, CS, HZ); Beijing Key Laboratory of Emerging Infectious Diseases (JH, HZ, YH, YL, CS, HZ); Division of Infectious Diseases, Beijing Ditan Hospital, Capital Medical University (HZ, NH); Institute of Basic Medical Theory, China Academy of Chinese Medical Sciences (YM); Division of 2nd In Vitro Diagnostic Reagents, National Institutes for Food and Drug Control (HZ); Department of Medical Laboratory, Beijing Tongren Hospital, Capital Medical University (XL); and Department of Geriatric Medicine, Beijing Tongren Hospital, Capital Medical University, Beijing, China (MQ).; ∗ These authors contributed equally to this study.

## Abstract

As cellular reservoirs, CD16^+^ monocyte subsets play important roles in the progression of HIV infection. Previous studies have shown that highly active antiretroviral therapy (HAART) reduced the percentages of CD14^high^CD16^+^ monocyte subsets, but did not recover the percentages of CD14^low^CD16^+^ subsets. Eighty-four chronic HIV-infected, HAART-naïve individuals and 55 HIV-negative subjects (31 without hyperlipidemia and 24 with hypertriglyceridemia) were enrolled. Plasma HIV-1 RNA levels, CD4^+^ T-cell counts, triglycerides, total cholesterol, high-density lipoprotein, and low-density lipoprotein were followed up for 48 weeks during HAART treatment in the longitudinal study. We found that mild hypertriglyceridemia in HIV-negative subjects and HIV-infected patients, naïve to HAART, did not affect the percentage of monocyte subsets. However, a failure of CD14^low^CD16^+^ subset recovery was observed in patients with HAART-related hypertriglyceridemia at 48 weeks. Thus, HAART-related hypertriglyceridemia altered homeostasis of monocyte subsets to antiviral therapy, which might further affect immune reconstitution.

## INTRODUCTION

Highly active antiretroviral therapy (HAART) has been proven to effectively suppress viral replication, decrease the total mortality of HIV-infected populations, and restore the immune function in HIV-infected patients.^1^ However, HAART cannot fully eradicate the virus because of the persistence of viral reservoirs. In additional to latent-infected memory CD4^+^ T cells,^2,3^ macrophages may comprise up to 10% of infected cells, and may contribute to the cellular reservoirs of the HIV virus,^4,5^ further leading to infection-related tissue disorders.^6^

As the progenitors of macrophages, peripheral blood monocytes are a heterogeneous population, consisting of classic CD14^high^CD16^−^, intermediate CD14^high^CD16^+^, and nonclassic CD14^low^CD16^+^ monocytes.^7,8^ Despite representing only 5–10% of circulating monocytes, CD16^+^ (CD14^high^CD16^+^ and CD14^low^CD16^+^) monocytes are important cellular targets for HIV-1 entry.^9^ CD14^high^CD16^+^ monocytes displayed an immunoregulatory phenotype, while CD14^low^CD16^+^ monocytes had a smaller size and granularity, and expressed higher levels of chemokine receptors CX3CR1,^7,10^ patrolled the endothelium of blood vessels, and were involved in the innate surveillance of local tissues.^7,8^

In our previous study, we observed significant differences between CD14^high^CD16^+^ and CD14^low^CD16^+^ monocyte sub sets with regard to their correlation with viral loads and responsiveness to antiviral therapy.^11^ Despite elevated percentages of both CD14^high^CD16^+^ and CD14^low^CD16^+^ monocyte subsets in HIV-infected HAART-naïve patients, antiviral therapy only successfully reduced the percentages of CD14^high^CD16^+^ monocytes, but increased the percentages of CD14^low^CD16^+^ monocytes.^11^

In addition to infection, the phenotype and percentage of monocyte subsets could be affected and regulated by other factors, such as hypercholesterolemia.^12^ With the progress of the disease and the exhaustion of CD4^+^ T cells, HIV-infected patients developed dyslipidemia, and mainly hypertriglyceridemia.^13,14^ In addition, almost all HAART drugs could alter the lipid levels to some degree and this caused hypertriglyceridemia, hypercholesterolemia, or mixed hyperlipidemia, especially Zidovudine (AZT), Stavudine (d4T), and most protease inhibitors (PIs).^15–17^ From 2011 to 2013, AZT/nonnucleoside reverse transcriptase inhibitor (NNRTI), d4T/NNRTI, and Tenofovir (TDF)/NNRTI were used by 51.2%, 11.2%, and 22.6% of patients, respectively, for the first line regimen in Asia.^18^ Although the regimen of HAART was gradually adjusted and improved, d4T and Nevirapine (NVP) were gradually declined and replaced, the regimen of AZT + 3TC (Lamivudine) + EFV (Efavirenz) for patients in the first-line ART was continued to be used extensively in Asia and many resource-limited areas.^19^

Here, we analyzed the effects of HAART-induced dyslipidemia on the recovery of peripheral monocyte subsets in HIV-infected patients and found that HAART-related hypertriglyceridemia was associated with an incomplete immune “recovery” (returning to the normal level) of CD14^low^CD16^+^ monocytes.

## METHODS

### Study Subjects, Follow-Up, and HAART

From 2010 to 2014, 84 chronic HIV-infected, HAART-naïve individuals (65 without hyperlipidemia, 18 with hypertriglyceridemia, and 1 with hypercholesterolemia) and 55 HIV-negative subjects (31 without hyperlipidemia and 24 with hypertriglyceridemia) were enrolled at Beijing Ditan Hospital and Beijing Tongren Hospital, Capital Medical University. This study was approved by the local ethics committee. All human blood samples were collected with informed consent, and the study conformed to the tenets of the Declaration of Helsinki.

HIV-infections were defined as described in the previous study.^11^ Blood samples were collected before initiating HAART, as baseline data, and the patients were followed up every 12 weeks for 48 weeks during HAART treatment. All patients were not concurrent infected with HCV, HBV, TB, and other infection. Samples were taken at about half a year postdiagnosis. For longitudinal studies, 65 chronic HIV-infected, HAART-naïve individuals with normal levels of serum lipids at baseline received antiviral regimens that consisted of 2 nucleotide reverse transcriptase inhibitors (NRTIs) and a nonnucleoside reverse transcriptase inhibitor (NNRTI), and were followed up for 48 weeks. Among 65 patients, 1 patient developed hypercholesterolemia, and 4 patients developed mixed hyperlipidemia at 48 weeks of follow-up. Thus, we analyzed 60 patients in the longitudinal study.

Plasma total cholesterol (TC), triglycerides (TG), high-density lipoprotein (HDL), and low-density lipoprotein (LDL) were regularly followed up at 24 and 48 weeks. Hyperlipidemia was defined according to the ATP III Guidelines of the National Cholesterol Education Program criteria (TC > 5.18 mmol/L, TG > 1.72 mmol/L).^20^

### Plasma HIV-1 Viral Load and CD4^+^ T-Cell Count

Plasma HIV-1 RNA levels and CD4^+^ T-cell counts were measured as described in previous studies,^11,21^ and both measurements were included in the National Quality Assurance Programs, twice per year.

### Immunophenotype Characterization of Monocytes

Whole blood from HIV-negative and positive subjects was collected for the monocyte subsets analysis by flow cytometry according to the previous study.^11^ The samples were performed within 4 hours. After red blood cell lysis, the following monoclonal antibodies were used: anti-human CD14, CD16, and CD45 (BD Biosciences, San Jose, CA). Matched isotype antibodies were used as negative controls. Monocytes were gated on the basis of forward scatter/side scatter characteristics and CD14/CD16 expression pattern. Samples were analyzed on FACS Calibur (BD Biosciences) using CellQuest software.

### Statistical Analysis

All statistical analyses were performed using SPSS version 14.0 software (SPSS Inc, Chicago, IL). Data were expressed as mean ± SD or mean ± SEM, as indicated. Flow cytometric data in different groups were analyzed using the one-way ANOVA test or independent *t* test. For all comparisons, *P* < 0.05 was considered to be statistically significant.

## RESULTS

### Baseline Characteristics and the Level of Serum Lipids Among Different Groups

**FIGURE 1:**
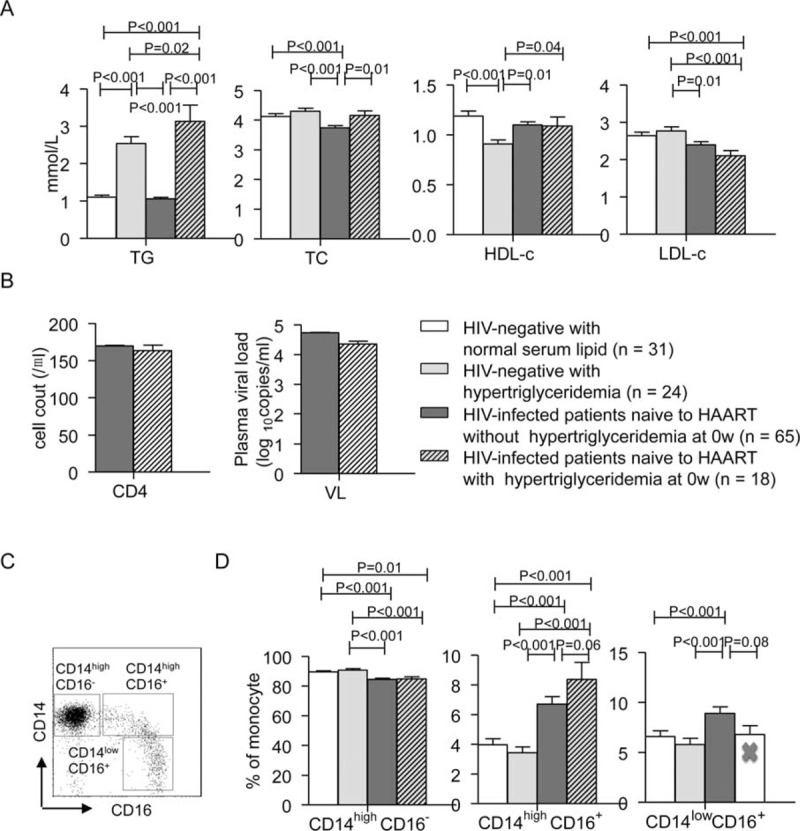
The serum lipid levels and the percentage of monocyte subsets of peripheral blood in 4 different groups. A, The serum TC (total cholesterol), TG (triglycerides), HDL-c (high-density lipoprotein cholesterol), and LDL-c (low-density lipoprotein cholesterol) levels among 4 different groups (HIV-negative subjects with normal lipid, N = 31; HIV-negative subjects with hypertriglyceridemia, N = 24; HIV-infected patients naive to HAART without hypertriglyceridemia, N = 65, and HIV-infected patients naive to HAART with hypertriglyceridemia, N = 18). B, The CD4^+^ T-cell counts and plasma HIV RNA levels in 2 groups (HIV-infected patients naive to HAART without hypertriglyceridemia, N = 65, and HIV-infected patients naive to HAART with hypertriglyceridemia, N = 18). C, Gating of monocyte subsets. Peripheral blood monocytes were divided into 3 subsets (CD14^high^CD16^−^, CD14^high^CD16^+^, and CD14^low^CD16^+^). D, The percentages of 3 monocyte subsets (CD14^high^CD16^−^, CD14^high^CD16^+^, and CD14^low^CD16^+^) among different groups were analyzed by flow cytometry within 4 hours. Data are shown as mean ± SEM of indicated patients. The data were analyzed using the one-way ANOVA test.

Eighty-four HIV-infected, naïve-to-HAART patients (65 without hyperlipidemia, 18 with hypertriglyceridemia, and 1 with hypercholesterolemia) were enrolled in our study. Demographic information, baseline characteristics, and the serum lipid levels are displayed in Table 1. For further investigation, we recruited 31 HIV-negative subjects with normal lipid levels, as well as 24 age-matched, HIV-negative subjects with hypertriglyceridemia (Table 1). The triglyceride levels in HIV-infected subjects with hypertriglyceridemia were higher than that of HIV-negative subjects with hypertriglyceridemia (2.54 ± 0.92 vs. 3.13 ± 1.88 mmol/L, respectively; *P* = 0.02; Table 1, Figure 1A). The total cholesterol levels in HIV-infected subjects with hypertriglyceridemia were lower than that in other groups (Figure 1A).

**TABLE 1:**
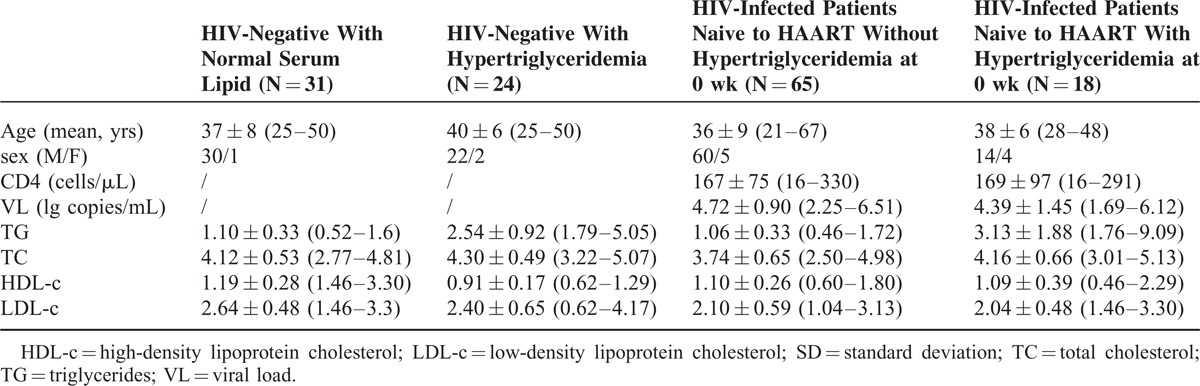
Characteristics of HIV-Negative Subjects With Normal Lipid, With Hypertriglyceridemia and HIV-Infected Patients Naive to HAART With or Without Hypertriglyceridemia and the Measurement of Serum Lipid (mmol/L), Mean ± SD (Range)

### Mild Hypertriglyceridemia Did Not Enhance the Percentage of Monocyte Subsets in HIV-Infected, HAART-Naïve Patients

We analyzed the percentages of 3 monocyte subsets (CD14^high^CD16^−^, CD14^high^CD16^+^, and CD14^low^CD16^+^ monocytes) in 4 groups of HIV-negative subjects with different levels of serum lipids (Figure 1A) by flow cytometry (Figure 1C), and did not find significant differences between HIV-negative with normal lipid level and with hypertriglyceridemia (Figure 1D), which indicated that mild hyperlipidemia did not influence the monocyte subsets in HIV-negative subjects.

Next, we analyzed the percentage of 3 monocyte subsets in HIV-infected patients, and HAART-naïve patients with or without hypertriglyceridemia. There was no difference in CD4^+^ T-cell counts and viral load between HIV-infected patients naive to HAART without and with hypertriglyceridemia at 0 weeks (Figure 1B). Consistent with the previous studies,^11^ an increased proportion of the CD14^high^CD16^+^ subset was observed in HIV-infected patients either with or without hypertriglyceridemia when compared with HIV-negative subjects with hyperlipidemia (Figure 1D). HIV-infected patients with hypertriglyceridemia did not exhibit increased levels of the CD14^low^CD16^+^ subset when compared with HIV-negative individuals with hypertriglyceridemia (Figure 1D).

### HAART Therapy-Related Hypertriglyceridemia Was Associated With a Failure of CD14^low^CD16^+^ Subset Recovery

It has been shown that some patients undergoing HAART might develop drug-related hypertriglyceridemia and/or hypercholesterolemia. Thus, we performed a longitudinal study of 65 patients with normal serum lipids who received HAART. At 48 weeks after receiving HAART therapy, among 65 patients, 17 (26%) patients developed hypertriglyceridemia, 1 patient developed hypercholesterolemia, and 4 patients (6%) developed mixed hyperlipidemia. Given the fact that the number of HIV-infected patients with hypercholesterolemia, we analyzed 60 patients in the longitudinal study. The regimen of HAART for HIV-infected patients without hyperlipidemia was summarized in Table 2.

**TABLE 2:**
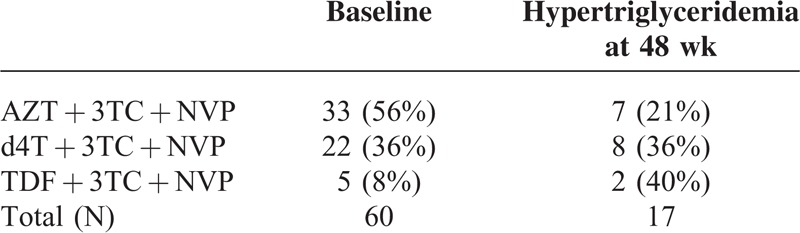
The Regimen of HAART for HIV-Infected Patients Without Hyperlipidemia, N (%)

**FIGURE 2:**
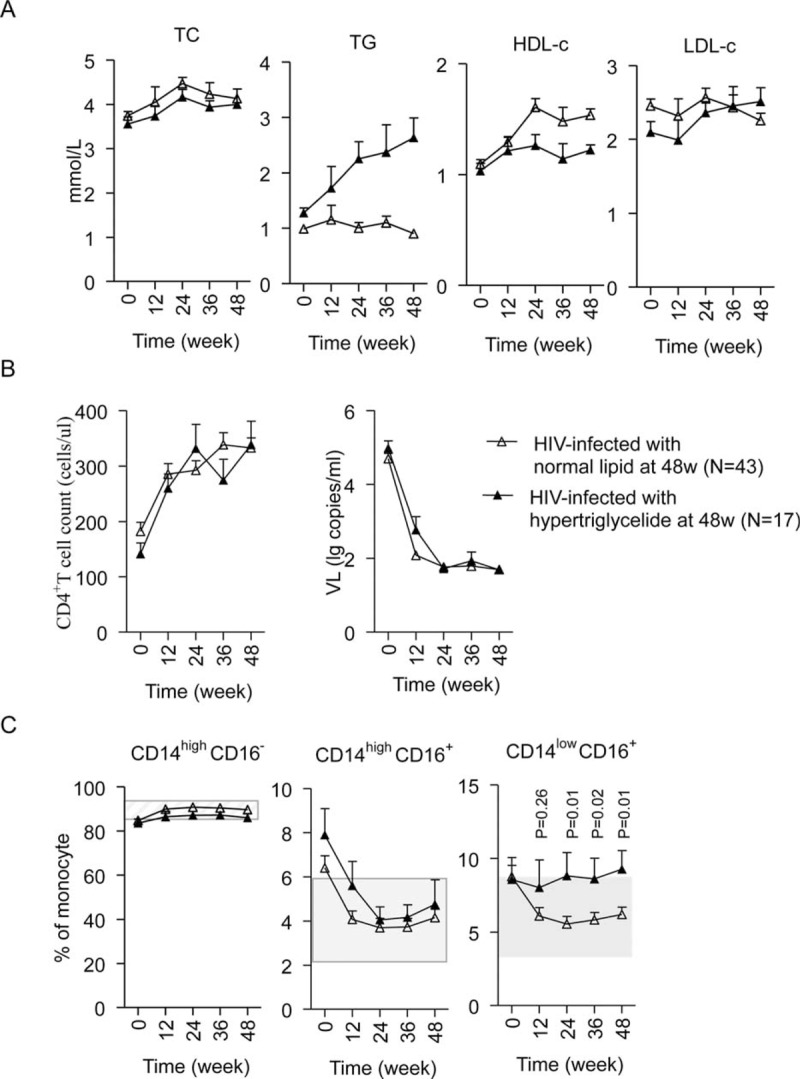
HAART-related hypertriglyceridemia blocked the recovery of CD14^low^CD16^+^ Subsets. A, Cross-sectional profiles of lipid (TC, TG, HDL-c, and LDL-c) levels, plasma HIV RNA levels, and CD4^+^ T cell counts. B, Kinetic changes of the proportion of 3 monocyte subsets. C, Two HIV-infected patients receiving HAART treatment for 48 weeks (hollow triangular: without hypertriglyceridemia at 48 weeks; filled triangular: with hypertriglyceridemia at 48 weeks) show a range like normal monocyte subsets. The difference of the CD14^low^CD16^+^ subsets between the 2 patients was analyzed using independent *t* tests.

Because of limited numbers of patients with hypercholesterolemia, we focused on the changes of monocyte subsets in patients with drug-related hypertriglyceridemia. Compared with patients with normal lipid levels (N = 43), the patients with hypertriglyceridemia exhibited higher levels of plasma triglycerides (from 1.28 ± 0.09 at 0 weeks to 2.63 ± 0.36 mmol/L at 48 weeks, N = 17), lower levels of HDL-c, and comparable levels of plasma total cholesterol and LDL-c (Figure 2A).

To explore whether hypertriglyceridemia affected the response of the patients to HAART, we analyzed the dynamic changes in viral loads and CD4^+^ T cell counts. Complete viral suppression in 2 groups was achieved and maintained in both groups within 24 weeks (Figure 2B). CD4^+^ T-cell counts increased gradually in 2 groups until reaching a plateau after 24 weeks of treatment (Figure 2B).

Consistent with our previous study, we observed that the proportion of circulating CD14^high^CD16^+^ monocytes declined gradually, which was in parallel with a slight increase in the proportion of CD14^high^CD16^−^ monocytes (Figure 2C) in both groups. Strikingly, HAART reduced the proportion of CD14^low^CD16^+^ monocytes in patients without hypertriglyceridemia after 48 weeks of follow-up. However, the patients with hypertriglyceridemia only showed a marginal decrease of CD14^low^CD16^+^ monocytes at 12 weeks of treatment and the percentages of CD14^low^CD16^+^ monocytes remained at a higher level during 48 weeks of treatment (Figure 2C). These results indicated that HAART-related hypertriglyceridemia might be associated with failure of recovery of CD14^low^CD16+ monocyte subsets.

## DISCUSSION

During the disease progression of HIV infection, CD16^+^ monocyte subsets act as cellular reservoirs for HIV replication as well as immunoregulatory cells for host inflammatory responses.^9,11,22^ In the previous study, we have reported an increase of CD14^high^CD16^+^ and CD14^low^CD16^+^ subsets in HAART-naïve patients. Strikingly, HAART only reduced the percentage of CD14^high^CD16^+^ monocytes, but not that of CD14^low^CD16^+^ subsets.^11^ In the present study with a longitudinal cohort, we showed a dramatic difference between the patients with normal serum lipids and those with HAART-induced hypertriglyceridemia with regard to the percentages of CD14^low^CD16^+^ subsets. Our data suggested that a failure of the recovery of the CD14^low^CD16^+^ subset was correlated with HAART-induced hypertriglyceridemia.

Because both infection and dyslipidemia might result in a disturbed homeostasis of monocytes,^11,12^ HIV virus and serum lipid levels should be taken into consideration when explaining the higher CD14^low^CD16^+^ monocyte levels in patients with HAART-induced hypertriglyceridemia. However, although both CD14^high^CD16^+^ and CD14^low^CD16^+^ subsets were elevated in HAART-naïve patients, CD14^high^CD16^+^ rather than CD14^low^CD16^+^ monocyte subsets have been shown to correlate with increased viral loads.^8,11^ Because viral suppression was achieved within 12 weeks in our cohort, regardless of serum levels of TG, the difference of the CD14^low^CD16^+^ subset between patients with normal TG and with hypertriglyceridemia appeared not to result from the different status of HIV replication.

A number of studies have shown a correlation between hyperlipidemia and monocyte subsets. It has been shown that patients with hypercholesterolemia displayed lower monocyte counts and higher percentage of CD14^low^CD16^+^ monocyte subsets.^12^ In the present study, the majority of the cases with HAART-related hyperlipidemia were mild hypertriglyceridemia (17/60, 28%). Because NVP was considered a “lipid-friendly” drug,^16^ hypertriglyceridemia was mainly induced by AZT and d4T in our study. In patients with familial hypercholesterolemia, we found a significant increase of CD14^high^CD16^+^ monocytes (our unpublished data). In the present study, the majority of the cases with HAART-related hyperlipidemia were mild. Comparable levels of triglycerides did not enhance the percentages of CD14^low^CD16^+^ monocytes either in HIV-negative subjects or in HIV-infected, HAART naïve patients. Thus, mild hyperlipidemia alone did not significantly affect the homeostasis of monocyte subsets. However, in our cohort receiving NRTIs and NNRTI-based regimens, similar levels of triglycerides were correlated with a failure of recovery of CD14^low^CD16^+^ monocytes, which indicated that anti-viral drug-induced defects in lipid metabolism might affect homeostasis of monocyte subsets via unknown mechanisms.

Long-term HIV and HAART exposure is associated with elevated risks for non-AIDS-related complications, including metabolic abnormalities, dyslipidemia, and cardiovascular disease.^16,17^ Accumulated studies have focused on the role of monocytes and macrophages in the progression of HIV or HAART-related cardiovascular diseases.^23,24^ Hypercholesterolemia has been shown to promote monocyte adherence, and further initiate the process of atherosclerosis.^25^ CD14^low^CD16^+^ monocytes could recognize viral products and adhere to the vascular endothelium via CX3CR1^7^ and be recruited into different tissues and differentiate into mature macrophages or dendritic cells. Thus, these cells might not only act as a persistent source of HIV, but also serve as local immune effectors and regulatory cells. Besides CX3CR1, CD14^low^CD16^+^ monocytes express higher levels of chemokine receptor CCR5, which is a coreceptor for HIV-1 entry and chemokine receptor for monocyte migration. It has been shown that maraviroc, an antagonist of CCR5, reduced the atherosclerotic progression by interfering with monocyte recruitment into plaques.^26^ Recently, higher levels of tissue factor and 62P were detected in CD14^low^CD16 ^+^monocytes in HIV-infected subjects, which indicated that this subset tended to home to the vascular endothelium and could become foam cells.^8,27^

In summary, our study explored a correlation between HAART-induced hypertriglyceridemia and a failure in recovery of CD14^low^CD16^+^ monocytes, which might be a novel mechanism affecting immune reconstruction in AIDS patients. In the current study, 17 among 65 patients developed hypertriglyceridemia and were analyzed to explore the effect of hypertriglyceridemia on monocyte subsets, and more patients should be recruited for the detailed mechanism in the future study. Because only NRTIs and NNRTIs were used in our cohort, whether PI-related dyslipidemia affected the recovery of monocyte subsets needs to be further investigated. Besides, given the fact that the number of cases was limited due to the limited numbers of hypercholesterolemia in our study, we did not reveal the role of hypercholesterolemia in the AIDS process. Moreover, due to the mildness of hyperlipidemia and adherence effects, our patients did not receive lipid-lowering drugs during 48 weeks of HAART. Recent studies have shown that rosuvastatin significantly reduced monocyte activation and the percentage of CD14^low^CD16^+^ monocytes.^28^ Thus in future studies, it will be of interest to investigate whether lipid-lowering drugs can reduce CD14^low^CD16^+^ monocyte subsets in HIV-infected patients receiving HAART.
